# A computational study of right ventricular mechanics in a rat model of pulmonary arterial hypertension

**DOI:** 10.3389/fphys.2024.1360389

**Published:** 2024-03-11

**Authors:** Oscar O. Odeigah, Ethan D. Kwan, Kristen M. Garcia, Henrik Finsberg, Daniela Valdez-Jasso, Joakim Sundnes

**Affiliations:** ^1^ Simula Research Laboratory, Oslo, Norway; ^2^ Shu Chien-Gene Lay Department of Bioengineering, University of California San Diego, La Jolla, CA, United States

**Keywords:** pulmonary arterial hypertension, right ventricle, cardiac mechanics, finite-element models, gradient-based optimization, data assimilation

## Abstract

Pulmonary arterial hypertension (PAH) presents a significant challenge to right ventricular (RV) function due to progressive pressure overload, necessitating adaptive remodeling in the form of increased wall thickness, enhanced myocardial contractility and stiffness to maintain cardiac performance. However, the impact of these remodeling mechanisms on RV mechanics in not clearly understood. In addition, there is a lack of quantitative understanding of how each mechanism individually influences RV mechanics. Utilizing experimental data from a rat model of PAH at three distinct time points, we developed biventricular finite element models to investigate how RV stress and strain evolved with PAH progression. The finite element models were fitted to hemodynamic and morphological data to represent different disease stages and used to analyze the impact of RV remodeling as well as the altered RV pressure. Furthermore, we performed a number of theoretical simulation studies with different combinations of morphological and physiological remodeling, to assess and quantify their individual impact on overall RV load and function. Our findings revealed a substantial 4-fold increase in RV stiffness and a transient 2-fold rise in contractility, which returned to baseline by week 12. These changes in RV material properties in addition to the 2-fold increase in wall thickness significantly mitigated the increase in wall stress and strain caused by the progressive increase in RV afterload. Despite the PAH-induced cases showing increased wall stress and strain at end-diastole and end-systole compared to the control, our simulations suggest that without the observed remodeling mechanisms, the increase in stress and strain would have been much more pronounced. Our model analysis also indicated that while changes in the RV’s material properties–particularly increased RV stiffness - have a notable effect on its mechanics, the primary compensatory factor limiting the stress and strain increase in the early stages of PAH was the significant increase in wall thickness. These findings underscore the importance of RV remodeling in managing the mechanical burden on the right ventricle due to pressure overload.

## 1 Introduction

Pulmonary arterial hypertension (PAH) is a medical condition marked by a persistent elevation in mean pulmonary arterial pressure (mPAP). The prolonged elevation in mPAP imposes a significant burden on the right ventricle, resulting in impaired right ventricular (RV) function which can lead to heart failure (Voelkel et al., 2012). The prognosis of the disease is notably grim, with a median survival time of merely 3–5 years post-diagnosis (Hurdman et al., 2012). Studies have highlighted the crucial role of RV function as a prognostic indicator for disease progression, severity, and patient survival (van Wolferen et al., 2007; Howard, 2011; Swift et al., 2017). The sustained pressure overload on the RV in PAH triggers geometric remodeling in the form of hypertrophy (Lamberts et al., 2007), altered myocardial contractility (Vélez-Rendón et al., 2018), and RV free wall stiffening (Rain et al., 2016). These remodeling mechanisms help to maintain cardiac output in the early stage of PAH, but can eventually lead to a decline in RV function in the later stages of the disease (Vonk Noordegraaf et al., 2017).

Despite the link between RV function and patient survival in PAH, there has been limited research attention given to RV remodeling compared to LV remodeling in systemic hypertension (Odeigah et al., 2022). This limited research has created a knowledge gap in regards to our understanding of how PAH-induced remodeling affects RV function. Studies have shown that concentric hypertrophy is associated with preserved RV systolic function in the early stages of PAH (Badagliacca et al., 2015). On the other hand, recent studies have shown that myocardial stiffening, the main contributor to increased RV diastolic stiffness (Kakaletsis et al., 2023), prevents pathological RV dilation in the early stages of PAH (Kwan et al., 2021), but can be associated with impaired RV relaxation and diastolic dysfunction as the disease progresses, making it an important prognostic indicator of disease severity Rain et al. (2013); Trip et al. (2015).

However, the specific alterations in RV mechanics induced by these remodeling mechanisms are still not well understood. As altered ventricular mechanics can impact myocardial perfusion leading to RV ischemia (Strauer, 1979; Alter et al., 2016), and can also cause right-to-left ventricular dyssynchrony (Vonk-Noordegraaf et al., 2013), it is clear that an understanding of how RV mechanics is altered by PAH-induced remodeling is of significant clinical interest. Furthermore, untangling the relative effects of geometric remodeling and altered material properties on RV mechanics can potentially unveil new and independent predictors of disease severity, or at the very least, provide insight into which mechanisms dominate RV mechanical response. Overall, there is a critical need to investigate the mechanical changes occurring in the RV during the progression of PAH and to understand the relative effects of the different remodeling mechanisms on RV mechanics.

The present study seeks to integrate experimental data from a rat model of PAH measured over a 12-week period into a computational model to elucidate RV mechanical changes during the progression of PAH. We confined our analysis to three time points that exhibited distinct hemodynamic remodeling phenotypes reported previously (Kwan et al., 2021). We aim to quantify the impact of geometric remodeling and changes in right-ventricular myocardium wall properties on chamber mechanics using predictions of wall stress and strain as indices. The objective is to develop a robust framework to investigate the effects of PAH-induced remodeling on RV mechanics.

## 2 Materials and methods

### 2.1 Data acquisition

Pulmonary arterial hypertension was induced in rats as described in (Kwan et al., 2021) using the well-established sugen-hypoxia (SuHx) model, an animal model that recapitulates vascular remodeling found in PAH patients (Abe et al., 2010). Male Sprague-Dawley rats (7 weeks old and weighing 214 ± 23 g) were administered with 20 mg/kg of sugen, a vascular endothelial receptor blocker, and kept in 10% O_2_ hypoxia for 3 weeks. The animals were then removed from the hypoxic chamber and returned to normoxia (21% oxygen) where the pulmonary arterial pressures continued to rise. Age-matched animals were kept in normoxia during the entire period to serve as the control group. The animals underwent invasive hemodynamic procedures at 4, 8, and 12 weeks post-SuHx induction. The three time points chosen to build three-dimensional biventricular models were based on a study of RV remodeling involving six time points along the disease progression (Kwan et al., 2021). Briefly, we found that after 4 weeks of sugen-hypoxia, rats had significant rise in end-systolic pressures but no changes in ejection fraction, attributed to significant RV hypertrophy. However, later in the disease or rats studied in later weeks of sugen-hypoxia, there were no more changes in hypertrophy. Instead, there was a sharp rise in end-diastolic pressure and end-diastolic elastance with preserved end-diastolic volume. By 12 weeks of sugen-hypoxia, animals show a small reduction in RV end-diastolic elastance, in ejection fraction, and a slight increase in volume. Here we sought to investigate these features. While we note a gradual decrease in the RV ejection fraction over 12 weeks (from 65% in the control to 50%, 47%, and 43% after 4, 8, and 12 weeks), the ejection fraction remained above heart failure thresholds of 35%–40% (Meyer et al., 2010).

Following previously described methods (Vélez-Rendón et al., 2018), all animals underwent invasive open-chest measurements of blood pressure and volume taken in the right and left ventricles while kept under 2.5% isoflurane. Pressure-volume (P-V) timeseries were aligned within the cardiac cycle and averaged. End of systole (ES) was determined by identifying the maximum pressure-to-volume ratio point in the P-V loop. The end-diastolic point was identified as the timepoint in the P-V loop where the pressure was at a minimum and the pressure rate of change (i.e., dp/dt) was at a maximum. After these hemodynamic measurements were taken, the heart was flushed and excised. RV free wall thickness measurements were taken *ex vivo* across the wall and averaged to obtain a representative RV wall thickness.

### 2.2 Biventricular shape model

Rat-specific meshes were built using data from hemodynamic pressure-volume timeseries and morphological measurements from harvested hearts from four different rats. These rats were selected to represent normotensive and three distinct sugen-hypoxia groups, published in Kwan et al. (2021). The idealized three-dimensional biventricular meshes were built using Gmsh (Geuzaine and Remacle, 2009) and each rat-specific model was parameterized based on the animal hemodynamic and morphological data outlined in Table 1.

**TABLE 1:** Right ventricular wall thicknesses and cavity volumes used to build the computational meshes.

Case	Wall thickness, mm	Cavity volume, *μ*L
Control	0.8	170
SuHx Week 4	1.6	187
SuHx Week 8	1.8	202
SuHx Week 12	1.9	308

LV wall thickness and cavity volume were fixed to 2 mm and 165 *μ*L respectively for all cases.

**FIGURE 1:**
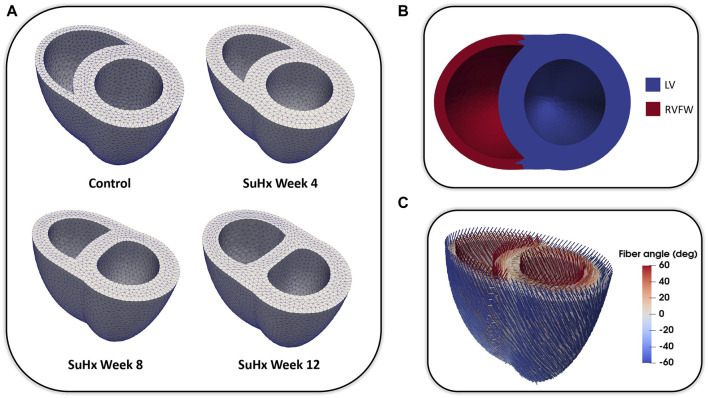
**(A)**: Idealized three-dimensional finite element meshes representing the control, SuHx Week 4, SuHx week 8, and SuHx week 12 cases. **(B)**: Control mesh partitioned into two regions representing the RVFW (in red) and the LV (in blue) which comprises the LVFW and septum. **(C)**: Myocardial fibers embedded in the control mesh using the Laplace Dirichlet Rule-Based algorithm. LV, left ventricle; LVFW, left ventricular free wall; RVFW, right ventricular free wall; SuHx, sugen-hypoxia.

We adapted the RV wall thickness of each mesh to match the experimentally measured RV wall thickness and inflated both ventricles to match the cavity volumes presented in Table 1. The meshes were then partitioned into two regions representing the right ventricular free wall (RVFW) and the LV region, which consisted of the left ventricular free wall (LVFW) and the interventricular septum. The morphology, myocardium properties and hemodynamics of the LV were kept constant for all cases, as there was no indication of changes in the left ventricle in any of these animals (Kwan et al., 2021). The muscle-fibre architecture was registered on the meshes using the Laplace Dirichlet Rule-Based algorithm (Bayer et al., 2012). We prescribed the myofiber helix angle to vary transmurally from +60 deg at the endocardium to −60 deg at the epicardium. In Figure 1, we show the meshes built for each case, along with an example of the mesh partitioning and myocardial fiber registration on the control case mesh.

### 2.3 Mathematical modeling

We represent the heart as a continuum body, where the coordinates in the reference configuration (***X***) are mapped to coordinates in the current configuration (***x***) via the deformation gradient:

F=I+∇u, (1)

with ***u*** = ***x*** − ***X*** denoting the displacement of a given point in the domain **Ω** (i.e., the myocardium). The displacement field is found by solving the quasi-static equilibrium equation given by:

∇⋅P=0, (2)

where ***P*** is the first Piola-Kirchhoff stress tensor, subject to imposed boundary conditions. The basal displacement of our biventricular domain was set to zero in the longitudinal (apex-to-outflow) direction. Basal movement in the other directions, as well as the movement of the epicardial surface, was restricted by a linear spring of stiffness *k* = 0.5 kPa/cm^2^ as in a previous study (Finsberg et al., 2018b). Measured LV and RV pressures were applied as Neumann boundary conditions at the endocardial surfaces of the domain.

To model the passive behavior of the myocardium, we used the transversely isotropic form of the hyperelastic strain energy function proposed in Holzapfel and Ogden (2009):

$$\Psi \left(F\right)=\frac{a}{2b}\left(e^{b\left(I_{1}-3\right)}-1\right)+\frac{a_{f}}{2b_{f}}\left(e^{b_{f}{\left(I_{4f_{0}}-1\right)}^{2}}-1\right),$$ (3)

where *a*, *a*_*f*_, *b*, *b*_*f*_ are material stiffness parameters, and the invariants are defined as:

$$I_{1}=trC,\;I_{4f_{0}}=f_{0}.\left({Cf}_{0}\right),$$ (4)

with ***C*** denoting the right Cauchy Green tensor and ***f***_0_ the myocardial fiber direction. We assume the myocardium is incompressible and enforce this by adding an extra term *p* (*J* − 1) to the strain energy function with *p* being a Lagrange multiplier representing the hydrostatic pressure, and *J* = det(***F***).

To model the active behavior of the myocardium, we applied the commonly used active stress formulation (Nash and Hunter, 2000):

$$\sigma =\sigma_{p}+\sigma_{a}$$ (5)

where ***σ*** is the total Cauchy stress tensor which is decomposed into a passive stress contribution:

$$\sigma_{p}=J^{-1}\frac{\partial \Psi }{\partial F}F^{T},$$ (6)

and an active stress contribution due to the contraction of cardiomyocytes:

$$\sigma_{a}=T_{a}\left[\left(f⊗f\right)+\eta \left(I-f⊗f\right)\right].$$ (7)

The magnitude of the active stress is denoted by *T*_*a*_ and *η* controls the amount of active stress developed in the directions transverse to the fiber direction, as studies have shown that active stresses in the transverse direction (i.e., sheet and sheet-normal directions) are non-negligible (Lin and Yin, 1998). Similar to the study by Sundnes et al. (2014), we have assumed homogeneity in transverse active stress and consequently set *η* to a fixed value of 0.2 (or 20%). Notably, the total Cauchy stress tensor ***σ*** and the first Piola-Kirchhoff stress tensor ***P*** are related by the expression ***P*** = *J* ***σ*** ^*T*^ ***F*** ^−*T*^.

### 2.4 Model calibration

The model was calibrated to match *in vivo* pressure-volume (P-V) time series data. Measured pressures were provided as input parameters to the model (endocardial boundary conditions), and the model parameters were adjusted until the calculated volumes agreed with the measured ones. The model-data volume mismatch was defined as

$${\left(\frac{V_{RV}^{i}-{\tilde{V}}_{RV}^{i}}{V_{RV}^{i}}\right)}^{2},$$ (8)

where ${\tilde{V}}_{RV}$ and *V*_*RV*_ are the simulated and measured RV cavity volumes, respectively, and *i* denotes a specific time point.

**FIGURE 2:**
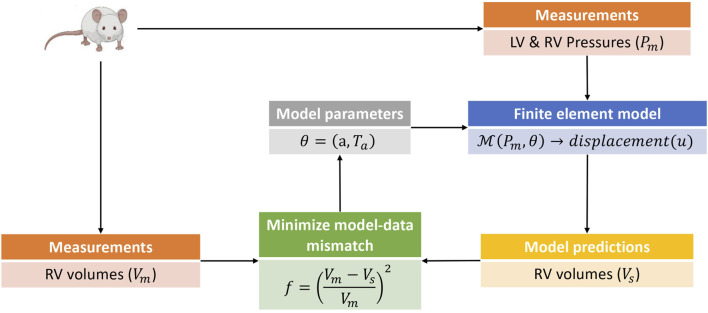
This is a schematic of the parameter estimation pipeline used in this study. The parameter estimation was done in two phases with an estimation of the isotropic scaling parameter *a* done in the first phase by fitting the model to passive filling pressure-volume data. The second phase involved an estimation of the active stress *T*_*a*_ by fitting the model to pressure-volume data from the active phases of the cardiac cycle. Only model predicted RV volumes were used in the parameter estimation pipeline, as the parameters and hemodynamics for the LV were held fixed for all simulation cases (see Section 2.1). The pressure-volume data was obtained from rats induced with PAH via the sugen-hypoxia (SuHx) protocol, as well as from a control rat. LV, left ventricle; RV, right ventricle.

Model calibration was carried out in two phases. In the first phase, the passive (isotropic) stiffness parameter *a* in (3) was estimated by fitting the model to P-V data in the passive filling phase of the cardiac cycle. Due to the sparsity of data used for the optimization, the remaining three material parameters in (3) were not estimated, but fixed to values (*b* = 5.0, *a*_*f*_ = 2.582kPa, and *b*_*f*_ = 5.0) from Finsberg et al. (2018b) for all simulations in this study. In the second phase, the optimized *a* parameter was held fixed at its fitted value from the first phase, and the active stress scaling parameter *T*_*a*_ in (7) was estimated by fitting the model to P-V data through the active phases of the cardiac cycle (i.e., isovolumic contraction, ejection and isovolumic relaxation). Since both the P-V values and muscle contraction varies throughout the cardiac cycle, *T*_*a*_ was allowed to vary in time, with a separate value estimated for each measured time point in the cardiac cycle. Figure 2 presents an overview of the parameter estimation pipeline.

### 2.5 Estimation of end-diastolic and end-systolic elastance

End-diastolic elastance (E_ed_) and end-systolic elastance (E_es_), proposed as global indices of ventricular stiffness (Templeton et al., 1972) and ventricular contractility (Suga and Sagawa, 1974) respectively, were estimated in this study. These metrics were estimated by perturbing the loading conditions on the optimized model while keeping all other variables fixed. Specifically, at the ED point, the ED pressure *P*_*ed*_ was perturbed by incrementing it with a factor (*P*_*ed*+Δ_ = *P*_*ed*_ + Δ*P*), resulting in a change in ED volume (*V*_*ed*+Δ_ = *V*_*ed*_ + Δ*V*). The estimate of ED elastance was then obtained by dividing the change in pressure by the change in volume, given as

$${\tilde{E}}_{ed}=\frac{\Delta V}{\Delta P},$$ (9)

where Δ*P* was set at 0.1kPa. The same approach was used for estimating E_es_ by perturbing the optimized model at the ES point.

### 2.6 Simulation and implementation details

To solve the set of partial differential equations described in (2), we implemented a Galerkin finite element method, which involved discretizing the variational form of (2) using Taylor-Hood tetrahedral finite elements (Hood and Taylor, 1974). Specifically, we used piecewise quadratic basis functions for the displacement field and piecewise linear basis functions for the hydrostatic pressure field. We used a previously developed cardiac mechanics software (Finsberg, 2019) implemented in the FEniCS finite element framework (Logg et al., 2012) to solve the numerical problem. For a detailed derivation of the variational form of (2), interested readers can refer to the work by Finsberg et al. (2018b).

For the minimization of the objective function (8) we used the Broyden-Fletcher-Goldfarb-Shanno (BFGS) algorithm (Broyden, 1970; Fletcher, 1970; Goldfarb, 1970; Shanno, 1970) implemented in the SciPy library (v 1.11.3) (Virtanen et al., 2020) in Python.

## 3 Results

### 3.1 Model calibration

**FIGURE 3:**
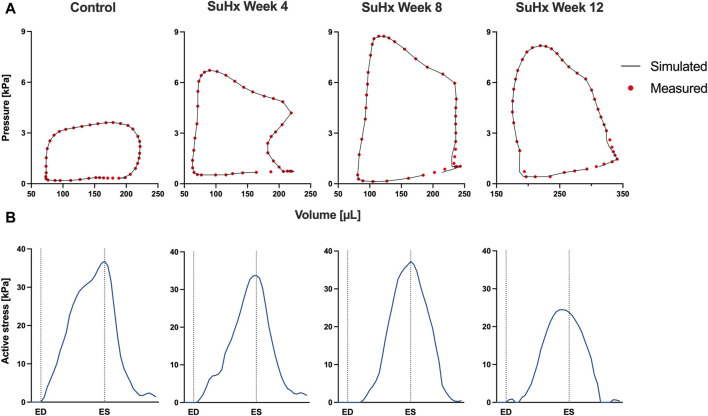
Model calibration results. **(A)** Model-predicted (black lines) and experimental (red circles) RV P-V loops for control, week 4, week 8, and week 12 SuHx cases, demonstrating a good fit between model and data. LV P-V loops are not displayed as the same LV data was used across all cases. **(B)** Time traces of the optimized active stress parameter *T*_*a*_ for each case. On the *x*-axis we plot the normalized time over one cardiac cycle. The vertical dotted lines indicate the timings of end-diastole (ED) and end-systole (ES). The active stress did not exhibit a distinct trend, likely influenced by specific modeling choices, discussed in Section 4.4.

**FIGURE 4:**
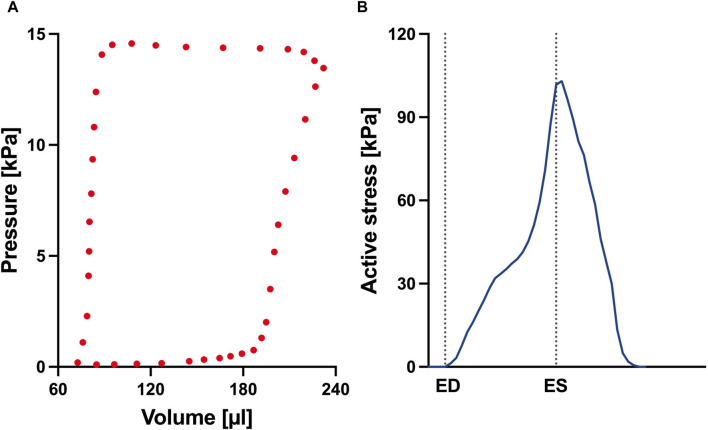
**(A)** Left ventricular pressure-volume loop used for all simulations in this study. **(B)** Time trace of the active stress parameter used in the active myocardium model for the left ventricle (LV). On the *x*-axis we plot the normalized time over one cardiac cycle. The vertical dotted lines indicate the timings of end-diastole (ED) and end-systole (ES). This curve was derived by fitting a biventricular model to left ventricular and right ventricular pressure-volume data from the control animal. The rationale for this approach is that given there are no significant changes to the LV during early-stage PAH, we can assume that the LV material remains at the normal (control) state.

The simulated and measured P-V loops for the RV are shown in Figure 3 for the different simulated cases. The results show a very good fit between our model and the experimentally measured data. Time traces of the optimized active stress parameter *T*_*a*_ are also presented in Figure 3. In addition, we present the optimized passive material parameter *a* for the control, week 4, week 8 and week 12 SuHx cases in Table 2. As previous studies have shown no significant change in LV hemodynamics in early-stage PAH (Kwan et al., 2021), the same P-V data and material parameters were used for the LV in all simulated cases (Figure 4; Table 2). The LV active and passive material parameters were calibrated using P-V data from the control animal.

**TABLE 2:** Optimized passive material parameter.

Case	LV passive material parameter, kPa	RV passive material parameter, kPa
Control	1.42 for all cases	0.07
SuHx Week 4		0.22
SuHx Week 8		0.23
SuHx Week 12		0.18

**FIGURE 5:**
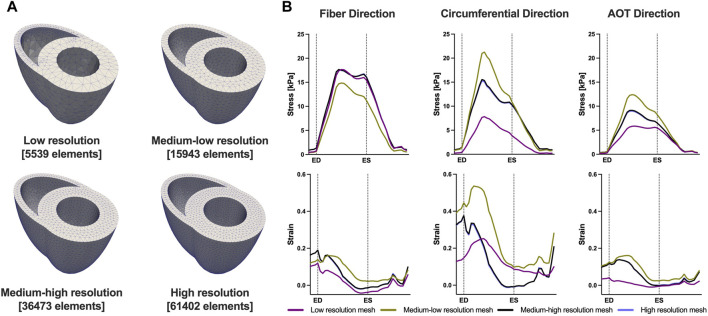
Results of the mesh convergence analysis. **(A)** Four distinct mesh resolutions for the control geometry used in the mesh convergence analysis. **(B)** Model predicted average Cauchy stress and Green strain from the four distinct mesh resolutions. On the *x*-axis we plot the normalized time over one cardiac cycle. The vertical dotted lines indicate the timings of end-diastole (ED) and end-systole (ES). Model predictions showed low sensitivity to mesh resolution beyond the Medium-high resolution threshold.

We conducted a mesh convergence analysis based on the control geometry and hemodynamics to find the optimal mesh resolution needed for accurate model predictions. Specifically, we calculated the average Cauchy stress and Green strain employing four distinct mesh resolutions, ranging from a low-resolution mesh comprising approximately 5,500 elements to a high-resolution mesh comprising approximately 61,000 elements. As depicted in Figure 5, the derived metrics exhibited a very low sensitivity to mesh resolution beyond the medium-high resolution (36,473 elements) threshold. This observation implies that a mesh resolution of 36,473 elements is adequate to ensure the accuracy of our model predictions. The chosen mesh sizes for the control, 4, 8, and 12 weeks post-PAH analyses are detailed in Table 3, including the respective average evaluation time for the cost functional (8), the number of cost-functional evaluations to fit one P-V point, and the total run times for each optimization process. All computational analyses were conducted on a computing cluster utilizing a single node with 32 cores.

**TABLE 3:** Mesh resolution, cost functional evaluation (eval) time, number of cost functional evaluations, and total run time of the optimization process for the different cases.

Case	No. of mesh elements	Functional eval time, seconds	No. of functional evaluations	Total run time, hours
Control	39 294	465.9 ± 236.2	6 ± 2	39
SuHx Week 4	43 995	596.5 ± 235.9	7 ± 2	52
SuHx Week 8	45 564	661.1 ± 287.4	7 ± 3	58
SuHx Week 12	49 910	895.3 ± 328.1	5 ± 2	64

Cost functional evaluation time and number of cost functional evaluations are average values for optimizing one measurement (i.e., pressure-volume) point of the data shown in Figure 3 along with standard deviations.

### 3.2 Mechanical analysis

#### 3.2.1 End-diastolic and end-systolic elastance

Table 4 presents the model-predicted end-diastolic elastance (E_ed_) and end-systolic elastance (E_es_) computed by perturbing RV pressure at the ED and ES points respectively, as described in Section 2.5. Additionally, we provide a comparison of the model-predicted values with the group means and standard errors from animals at the corresponding time points in Kwan et al. (2021). It should be noted that Kwan et al. only included animals up to 10 weeks post-PAH induction in their study. As such, the group means depicted in Table 4 for week 12 are actually for week 10 animals.

**TABLE 4:** Model-predicted end-diastolic (E_ed_) and end-systolic (E_es_) elastance compared with published group means ± standard error (SE).

Case	E_ed_ (group mean ± SE), mmHg/*μ*L	E_es_ (group mean ± SE), mmHg/*μ*L
Control	0.03 (0.017 ± 0.002)	0.29 (0.30 ± 0.033)
SuHx Week 4	0.06 (0.036 ± 0.01)	0.52 (0.39 ± 0.08)
SuHx Week 8	0.11 (0.13 ± 0.02)	0.55 (0.85 ± 0.13)
SuHx Week 12	0.13 (0.06 ± 0.02)	0.26 (0.76 ± 0.1)

Group means ± SE, shown for SuHx Week 12 are for week 10 animals, because the published study did not include week 12 animals.

Our model results reveal a consistent increase in E_ed_ from the control to week 12, while E_es_ demonstrates an upward trend from the control to week 8, followed by a decrease by week 12. This E_es_ trend aligns with the observations by Kwan et al. (2021). However, they observed a decrease in E_ed_ after week 8 which was not evident in our model results.

#### 3.2.2 Myocardial wall stress and strain

**FIGURE 6:**
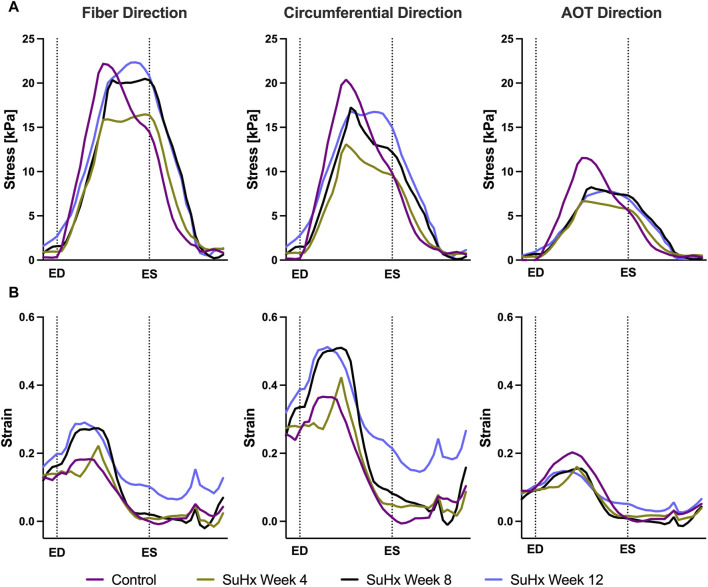
Model predictions of right ventricular free wall stress **(A)** and strain **(B)** in the fiber, circumferential, and apex-to-outflow (AOT) directions for the different cases simulated. On the *x*-axis we plot the normalized time over one cardiac cycle. The vertical dotted lines indicate the timings of end-diastole (ED) and end-systole (ES). Average stress and strain at ED and ES consistently increased in disease cases compared to the control. However peak systolic stress was higher in control compared to disease, while peak strain showed an opposite trend, being generally higher in disease compared to control.

Time traces of average Cauchy stress and Green strain in the RVFW along the fiber, circumferential, and apex-to-outflow (AOT) directions are shown in Figure 6 for the different simulated cases. To facilitate the direct comparison of model predicted stress and strain between the different cases, we aligned the pressure-volume (PV) data points within the cardiac cycle in such a way that an equal number of data points were consistently represented from ED to ES for each animal. For this reason, the *x*-axis in Figure 6 represents normalized time rather than actual time within a cardiac cycle. This approach allowed us to eliminate the expected variations in the timing of ED and ES across the different animals while enhancing the clarity of the comparison between them.

The results indicate that wall stress is highest along the fiber direction and lowest in the AOT direction, in both control and disease cases. At ED and ES, average RVFW stress monotonically increases from control to week 12. However, peak systolic stress, which occurred at the peak systolic pressure, was consistently higher in control compared to the disease cases except for week 12. In this case, peak systolic fiber stress was comparable with - and even slightly exceeded - that of the control animal (Table 5). This result can be explained by the considerable increase in passive stress at week 12 compared to control, despite a reduced active stress at week 12 (Figure 3B). We note that the total Cauchy stress in (5) is a sum of the passive and active stress components, as defined in 6, 7 respectively. The passive stress component is heavily influenced by changes in the mechanical properties of the myocardium, such as stiffness. The isotropic stiffness parameter is approximately 2.5 times greater at week 12 compared to control (Table 2). Similarly, in comparison to control, ED pressure and ED volume are about 4 times and 1.75 times greater, respectively, at week 12 (Figure 3A). These changes result in higher wall tension, thus contributing to the elevated passive stress. Hence, these mechanisms combined with our results of ED stress (Table 5), which gives an indication of passive stress and is at least 7.5 times higher in the fiber direction at week 12 compared to control, explain why the peak systolic fiber stress at week 12 is comparable to that at control, despite a diminished active stress at week 12.

**TABLE 5:** Model predictions of average right ventricular free wall stress and strain.

	Control	SuHx		
		Week 4	Week 8	Week 12
ED fiber stress, kPa	0.36	0.95	1.58	2.70
ES fiber stress, kPa	14.48	16.38	20.33	20.78
Peak fiber stress, kPa	22.18	16.46	20.47	22.34
ED circumferential stress, kPa	0.20	0.80	1.53	2.80
ES circumferential stress, kPa	9.84	9.59	12.19	14.98
Peak circumferential stress, kPa	20.39	13.08	17.24	16.80
ED AOT stress, kPa	0.02	0.39	0.70	0.98
ES AOT stress, kPa	5.59	5.76	7.30	6.93
Peak AOT stress, kPa	11.54	6.65	8.23	7.81
ED fiber strain	0.14	0.14	0.16	0.20
ES fiber strain	0.0002	0.01	0.02	0.10
Peak fiber strain	0.18	0.22	0.27	0.29
ED circumferential strain	0.27	0.28	0.34	0.39
ES circumferential strain	0.01	0.05	0.08	0.21
Peak circumferential strain	0.36	0.42	0.51	0.51
ED AOT strain	0.10	0.09	0.09	0.11
ES AOT strain	0.008	0.016	0.008	0.05
Peak AOT strain	0.20	0.16	0.15	0.15

Abbreviations: ED, end-diastole; ES, end-systole; AOT, apex-to-outflow; SuHx, sugen-hypoxia.

On the other hand, variations in wall strain between the control and disease cases were generally less pronounced than the stress variations, but showed an increasing trend with PAH progression at ED and ES, as shown in Table 5. In contrast to peak stresses, peak strains, specifically in the fiber and circumferential directions, were higher in disease compared to control which can be partly explained by the larger RV chamber volumes in the disease cases (Figure 3) leading to larger wall stretching. Peak AOT strain however, stayed within normal (control) values during the 12 weeks time course of this study.

**FIGURE 7:**
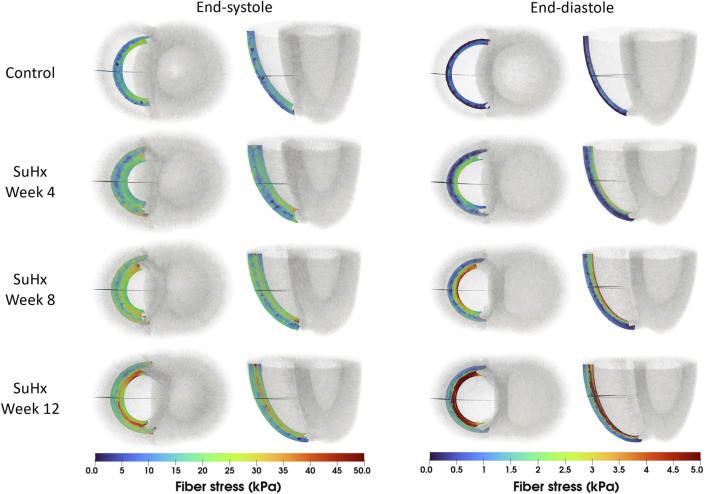
Spatial representation of fiber stress distribution at end-diastole (ED) and end-systole (ES) for the different simulated cases. Shown on axial and lateral slices in the middle of the right ventricular free wall, transmural stress variation increased with PAH progression, consistently higher in endocardial than epicardial regions.

The spatial distribution of stress in the RVFW is also presented in Figure 7, which enables us to observe the transmural variation of RVFW stress at ED and ES. Only small transmural variations in fiber stress were found for the control case. However, the transmural variation of stress becomes more pronounced as PAH progresses - especially at ED - with the endocardial regions consistently experiencing higher stress levels compared to epicardial regions.

#### 3.2.3 Effect of geometric and material remodeling on RV mechanics

To understand how the RV adapts in PAH, we simulated the effects of changes in pressure with no geometric nor myocardium wall properties changes, with only geometric changes, and with only changes in myocardium wall properties. Geometric changes were based on measured wall thickness (Table 1), while the wall material changes were based on passive and active material parameters fitted to pressure-volume data (Table 2; Figure 3B). It should be noted that while geometric changes include both increased wall thickness and increased RV radius (as a result of increased ED volume), in this analysis we limited it to increases in wall thickness. Given that the increased chamber radius is generally associated with increased stress, our results indirectly include this effect.

**FIGURE 8:**
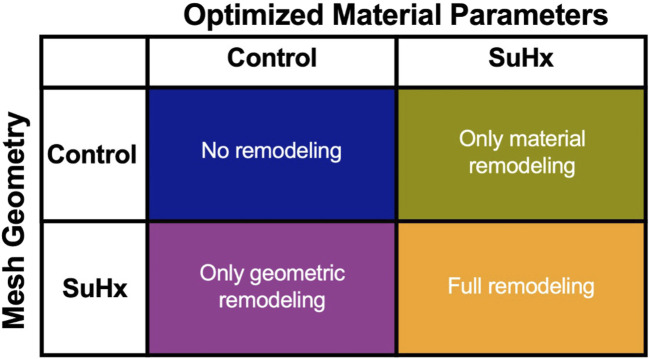
The set of simulations used to study the effect of geometric and material remodeling on RV mechanics by combining optimized material parameters and mesh geometries from control and SuHx (sugen-hypoxia) cases. Three theoretical combinations were considered: ‘no remodeling’ (control mesh and control material), ‘only material remodeling’ (control mesh and SuHx material) and ‘only geometric remodeling’ (SuHx mesh and control material). For all combinations, the geometric remodeling was quantified by the measured wall thickness (Table 1), the material remodeling by the optimized passive and active material parameters (Table 2; Figure 3B), while the hemodynamic data was fixed to that of the disease case in question. The ‘full remodeling’ case (SuHx mesh and SuHx material) is only added for comparison and results for this simulation have been presented previously in Table 5.

Figure 8 illustrates the three different theoretical cases with varying combinations of remodeling considered in this investigation. The fourth ‘full remodeling’ case shown in the figure is included here for comparison, as the stress and strain results for this case have been provided previously in Table 5. We also compared the results of these theoretical simulations with the control scenario (Table 5) to provide some context on how these parameter combinations leading to theoretical forms of remodeling impacted RVFW stress and strain.

**FIGURE 9:**
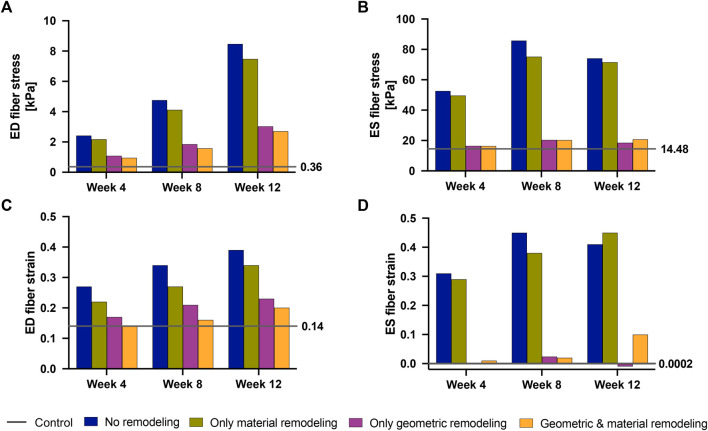
End-diastolic fiber stress **(A)**, End-systolic fiber stress **(B)**, End-diastolic fiber strain **(C)** and End-systolic fiber strain **(D)** at 4 weeks–12 weeks post-PAH induction when considering no remodeling (blue bars), only material remodeling (green bars) and only geometric remodeling (purple bars). For reference, the corresponding stress and strain values for the fully remodeled cases (orange bars) and for the control case (gray lines) are plotted. The control values are also called out to the right-hand side of each plot.

Our findings (Figure 9) revealed that geometric remodeling in the form of wall thickening plays a crucial role in moderating the increase in fiber stress and strain due to increasing pressure overload in PAH. In the absence of geometric remodeling, RVFW fiber stress and strain significantly increased, even to the level of cases with no remodeling at all. Notably, the addition of geometric remodeling alone had a much greater influence on end-systolic stress compared to end-diastolic stress, because it was almost sufficient to return end-systolic stress to normal. On the other hand, the absence of material remodeling had a much smaller, but not insignificant impact on these metrics, suggesting that the stress and strain response of the RVFW to pressure overload within the 12-week time course of this study is dominated by geometrical remodeling.

## 4 Discussion

In this study, we analyzed the right-ventricular mechanical changes due to pulmonary arterial hypertension by incorporating measurements from a sugen-hypoxia rat model into a computational biventricular model. We selected the sugen-hypoxia rat model as it is the smallest animal model to recapitulate vascular lesions resembling those found at autopsy in patients with PAH along with consequent ventricular remodeling (Taraseviciene-Stewart et al., 2001; Abe et al., 2010; Al-Husseini et al., 2015; Drozd et al., 2017; Jayasekera et al., 2020). The experimental data used in this study included animals up to 12 weeks post-PAH induction, and provided a representative snapshot of different disease stages, including a baseline control case.

Over the 12-week time course, myocardial stiffness increased by more than 100%, as indicated by end-diastolic elastance. Additionally, myocardial contractility, indexed by end-systolic elastance, almost doubled by week 8 compared to control, which was then followed by a decrease to within the baseline range by week 12.

Both end-diastolic and end-systolic stress and strain in the fiber, circumferential and apex-to-outflow directions consistently increased with disease progression; peak strain within the cardiac cycle exhibited a similar increase, except in the peak apex-to-outflow strain which remained within baseline limits. A contrasting trend was observed in peak systolic stress, occurring at the peak systolic pressure time point within the cardiac cycle. Our findings indicated that peak systolic stress was consistently higher in the control case, except for the peak systolic fiber stress at week 12, which marginally exceeded control. Overall, transmural stress variation across the RVFW was notably more pronounced in disease, with endocardial regions experiencing higher stress levels compared to epicardial regions of the RVFW.

Finally, we analyzed the relative effects of geometric and material remodeling on RV mechanics indexed by wall stress and strain. The simulations indicated that changes in heart geometry, particularly through wall thickening, had a more pronounced impact on moderating wall stress and strain than did changes in the material properties, such as wall stiffness. Notably, the influence of wall stiffening was pronounced at end-diastole, suggesting that the effect of the significant increase in myocardial stiffness on RVFW stress and strain was not negligible. Still, over the 12-week time course of this study, our results suggest that the stress and strain response of the RV was dominated by wall thickening.

### 4.1 Ventricular stiffness and contractility

End-diastolic elastance (E_ed_) and end-systolic elastance (E_es_) serve as valuable indices for evaluating ventricular stiffness and contractility, respectively (Templeton et al., 1972; Suga and Sagawa, 1974). The gold standard for computing these metrics is by transiently varying RV preload, generating multi-beat pressure-volume loops subsequently used for E_ed_ and E_es_ measurements (Suga et al., 1973; Maughan et al., 1979). This method, while also applied in clinical studies (Dell’Italia and Walsh, 1988; Hsu et al., 2020), is less established in the clinical setting compared to experimental studies. Here, we implemented a different approach that is comparable to the gold standard. We altered RV loading conditions by perturbing end-diastolic and end-systolic pressure, keeping all other parameters fixed. We then computed E_ed_ and E_es_ as the slope of the resulting pressure-volume relationship, i.e., ΔP/ΔV. This approach has been used previously for calculating E_es_ (Finsberg et al., 2018a; Finsberg et al., 2018b). We observed that the model predicts a slight increase in diastolic stiffness from week 8 to week 12, although the material stiffness parameter displayed in Table 2 is reduced. This apparent inconsistency is most likely the result of the increased wall thickness at week 12, which directly impacts the overall chamber elastance.

Nevertheless, the general trend of elevated stiffness in disease compared to control aligns with previous measurements in sugen-hypoxia (Kwan et al., 2021), monocrotaline (Vélez-Rendón et al., 2018), and pulmonary artery banding (Rain et al., 2016) animal models of PAH. Table 4 displays results from the study by Kwan et al. (2021), supporting this general trend. Likewise, Rain et al. (2016) demonstrated increased RV stiffness in rats with mild and severe RV dysfunction, attributing the rise in mild dysfunction to myofibril-mediated stiffness and in severe dysfunction to both increased myofibril stiffness and fibrosis. In a monocrotaline rat model, Vélez-Rendón et al. (2018) reported stiffening of the passive myocardium after 4 weeks post-PAH induction, observing an initial decrease in stiffness at week 1, possibly due to a temporary increase in myocardial compliance required to preserve RV stroke volume. However, by 2 weeks post-PAH induction, passive stiffness began trending upward. Our results also align with human clinical (Rain et al., 2013; Trip et al., 2015; Hsu et al., 2018) and computational (Finsberg et al., 2019) studies of PAH. In all four studies, they noted a progressive increase in RV passive stiffness in PAH patients compared to controls. This increase was found to be influenced by the degree of remodeling or the severity of the disease.

In our study, we observed an initial twofold increase in contractility from control to week 8, followed by a decrease at week 12, indicating a downregulation of RV contractility by 12 weeks post-PAH induction. This initial increase in contractility, reported in both animal (Blaudszun and Morel, 2012; Vélez-Rendón et al., 2018; Kwan et al., 2021) and human (Rain et al., 2013; Hsu et al., 2018; Finsberg et al., 2019) PAH studies, is linked to adaptive hypertrophy (Bogaard et al., 2009; Kwan et al., 2021), believed to preserve systolic function in early PAH stages (Naeije et al., 2014; Naeije and Manes, 2014). However, increased ventricular contractility is unsustainable, and a downregulation of contractile force is commonly observed as the disease progresses (Fan et al., 1997; Bogaard et al., 2009). Our model results indicate that this downregulation starts after 8 weeks post-PAH induction in a SuHx-rat model. Few PAH studies have explored the time course of contractility changes; most only distinguish between control and PAH subjects. In a relevant study, Finsberg et al. (2019) noted a 20% initial increase in RV contractility in mild RV remodeling, later down-regulating in severely remodeled cases to values below the control. Their study, focusing on human subjects, did not specify the time-course of the significant decrease in contractility but distinguished between mild and severe remodeling based on RV end-diastolic volume to LV end-diastolic volume ratios. Similarly, Kwan et al. (2021) reported downregulation at 10 weeks post-PAH induction in a sugen-hypoxia animal model, with values still higher than control at the 10-week timepoint. This suggests a peak in RV contractility at 8 weeks post-PAH induction, followed by a progressive downregulation, aligning with our observations.

### 4.2 Myocardial wall stress

Ventricular wall stress, particularly at end-systole (ES) and end-diastole (ED), plays a pivotal role in systolic and diastolic cardiac function. Its significant correlation with myocardial oxygen consumption and adverse cardiac remodeling has been well-established by previous studies (Strauer, 1979; Bogaard et al., 2009; Alter et al., 2016; Haque and Wang, 2017). Pressure overload directly impacts wall stress, adhering to the law of Laplace, and an elevation in wall stress can hinder myocardial oxygen supply by compressing coronary circulation (Chin et al., 2005). Consequently, deviations from normal or baseline wall stress can detrimentally affect oxygen availability to cardiomyocytes, potentially leading to cardiac ischemia, adverse remodeling, and ultimately, heart failure.

The inverse linear correlation identified by Quaife et al. (2006) between ES wall stress and RV ejection fraction, supported by Alter et al. (2016) for the LV, emphasizes the critical relationship between wall stress and cardiac function. This underscores the potential of wall stress as a diagnostic index for evaluating heart performance in disease. However, the lack of a direct method to measure RV wall stress necessitates reliance on mathematical approximations, with several proposed methods in the literature and no universally recognized gold standard. Consequently, the accuracy and agreement of these approximations carry significant experimental and clinical implications.

Our model results demonstrate a progressive increase in ED and ES fiber stress in disease compared to control. Circumferential and apex-to-outflow stress also exhibit an increase at ED and ES. Comparison with existing literature that reported wall stress in PAH, consistently revealed increased RV wall stress at ED and ES in disease compared to control (Quaife et al., 2006; Vélez-Rendón et al., 2018; Gold et al., 2020; Kwan et al., 2021), despite variations in geometric model assumptions and stress computation methods across these studies. Vélez-Rendón et al. (2018) and Kwan et al. (2021) assumed a spherical RV geometry and used the thin-walled Laplace law to estimate fiber stress in the RV free wall. Quaife et al. (2006) also assumed a spherical RV but employed a modified Laplace law suitable for non-circular cross-sections (Janz et al., 1989). Gold et al. (2020) utilized a two-dimensional model based on short-axis echo images and estimated wall stress using the von Mises formula, providing an equivalent stress over the entire RV rather than individual stress components. Finsberg et al. (2019), utilizing a modeling technique and stress approximation method similar to our study, did not report ED and ES wall stress, but instead reported peak fiber stress, which does not necessarily coincide with end-diastole or end-systole. Interestingly, they reported an increase in peak fiber stress only in severely remodeled cases which they defined as a ratio of RV ED volume (RVEDV) to LV ED volume (LVEDV) greater than 1.5. In the mildly remodeled case (RVEDV/LVEDV ≤1.5), peak fiber stress was at control level. This finding aligns with our observation that peak fiber stress, which occurred at a time point between ED and ES in our model, only surpassed control values at 12 weeks post-PAH induction, although it exhibited an increasing trend in the disease cases (Figure 6). This suggests that peak fiber stress increases as PAH progresses, and RV geometric remodeling transitions from wall thickening to dilation.

### 4.3 Effect of geometric and material remodeling

At the onset of PAH, both geometric and material remodeling occur simultaneously, and manifest as compensatory mechanisms. Our study aimed to isolate these remodeling mechanisms and explore their individual impacts on RV mechanics, as indexed by wall stress and strain. The results revealed that material remodeling has a minimal effect on wall stress and strain, while geometric remodeling plays a predominant role in reducing these parameters as the disease progresses. This agrees with existing knowledge that attributes the primary role of geometric remodeling to the reduction of wall stress (to normal physiological values) in the presence of pressure overload (Grossman et al., 1975).

Our study provides quantifiable insights into these established findings. Specifically, we found that if the RV wall had not thickened, ED fiber stress would have exhibited a substantial increase, reaching six-fold by week 4, eleven-fold by week 8, and a remarkable twenty-fold increase by week 12 compared to baseline (control) levels. However, due to observed geometric remodeling, the maximum increase in ED wall stress was limited to seven-fold, occurring at week 12. Similarly, ES wall stress would have experienced a three-fold increase by week 4 and a five-fold increase at weeks 8 and 12 without RV wall thickening. Again, the presence of RV wall thickening significantly mitigated these increases, with ES wall stress nearly fully normalized by week 12.

From these findings, two key takeaways emerge. Firstly, while geometric remodeling alone fell short of fully normalizing wall stress in the 12 weeks time course of this study, it effectively mitigated the majority of stress increases associated with rising RV afterload. Secondly, the presented magnitudes of wall stress increase, in the absence of RV thickening, shed light on what can be expected when RV geometric remodeling transitions from wall thickening to wall thinning in the later stages of PAH (in an attempt to maintain stroke volume and cardiac output). These excessive wall stress levels may lead to a detrimental sequence of cardiac ischemia, further compromised RV contractility, causing additional RV dilation, and ultimately culminating in RV dysfunction.

### 4.4 Limitations and future research directions

There are a number of important limitations to the present study, which should motivate further experimental and computational research. We applied the computational framework to a limited cohort of four animals, which included one normotensive and three hypertensive animals at distinct time points. Although the animals chosen for this study were representative of the control and disease groups, they do not represent a full description of the longitudinal and progressive remodeling of the RV in PAH. While not comprehensive, the study aimed to analyze mechanical changes in the RV during the initial 12 weeks post-PAH induction. Future research could apply the framework to a larger cohort over an extended period for a better understanding of RV behavior in PAH progression.

We only fit a single passive material parameter, while the others are set to values from the literature. This modeling decision was necessitated by the limited data available for the optimization. Attempting to fit all four parameters in (3) to *in vivo* P-V data would have substantially increased the computational cost. More importantly, it would have resulted in a scenario where multiple sets of parameters would minimized the cost function (8), rendering the chosen optimal parameter set overly sensitive to the initial guess provided to the optimization algorithm. While our approach aligns with previous research suggesting that a unique solution can be achieved by optimizing a single parameter (or at most two of the parameters) of (3) when fitting to *in vivo* P-V data (Hadjicharalambous et al., 2015; Balaban et al., 2017), it is crucial to recognize its inherent limitations, specifically the dependence on parameter values sourced from the literature. More detailed experimental data, for example, local ventricular strains estimated from magnetic resonance images, or biaxial stress-strain data extracted from myocardial tissue mechanical testing will be important for more accurate characterization of material properties, as well as for validation of the model results. As shown in Figure 3, the passive filling phase - which is the part used for optimizing *a*_*RV*_ - is nearly flat for several of the P-V loops used in this study. As such, small deviations in the pressure can lead to large variations in the fitted parameters. In addition, our decision to fit the isotropic material parameter in 3, while keeping the parameter that describes stiffness in the fiber direction, *a*_*f*_, constant in both control and disease cases, may have contributed to the absence of a discernible trend in active stress development, as depicted in Figure 3B. To address these issues, future model development should be based on more comprehensive datasets which should enable improved characterization of the passive tissue stiffness.

Due to the unavailability of imaging data of sufficient resolution to build meshes for the rats used in this study, we employed idealized biventricular geometries in the mechanical model. Despite this limitation, the idealized geometry gives insights on the effect of wall thickening on RV mechanics with PAH progression. It is important to note that the altered septal wall positions depicted in Figure 1 as the disease progresses stems from the methodology used in generating the biventricular meshes. Specifically, it is a result of the inflation of the mesh geometries to match the initial cavity volumes outlined in Table 1. While the magnitude of septal wall flattening was not validated against imaging data, qualitatively, it is consistent with cardiac magnetic resonance imaging data obtained from a different rat at time points similar to those used in this study.

We modeled the myocardium as transversely isotropic. The passive myocardium is inherently complex, displaying strong non-linearity and anisotropy, and would require an orthotropic model to fully characterize its mechanical behavior. However, it has been documented that a transversely isotropic formulation of the Holzapfel-Ogden law strikes a good balance between parameter identifiability–the ability to determine a unique parameter set given limited amount and quality of the data–and model fidelity–the ability of the model to adequately represent cardiac deformation and function (Gjerald et al., 2015; Hadjicharalambous et al., 2015). Given that the data available for model fitting in this study was quite limited, and the extensive use of a transversely isotropic model to approximate orthotropic cardiac tissue properties in the literature (Guccione et al., 1991; Xi et al., 2011; Balaban et al., 2017; Finsberg et al., 2018b; Avazmohammadi et al., 2019), we deemed the use of this model to be appropriate.

We did not include rat-specific measurements of fiber orientation or regional strain. Instead, we implemented the same fiber orientation across all simulations using a rule-based method and assumed a transmural variation in the fiber direction. This modeling choice is a candidate for further refinement as some studies have reported changes in myofiber architecture in PH (Avazmohammadi et al., 2017; Mendiola et al., 2023). However, it remains unclear to what degree myofiber angle remodeling occurs and whether previous studies are confounded by the animal model, or the stage of disease. In addition, our assumption of transmural (through-thickness) variation in the fiber direction is a common assumption in computational studies of cardiac mechanics that employ a rule-based method to assign myocardial fibers to the mesh geometry and has been validated by histology (Streeter Jr et al., 1969; Hill et al., 2014) and DTI-based studies (Hsu et al., 1998; Holmes et al., 2000; Scollan et al., 2000) of cardiac fiber architecture. Nevertheless, future research could benefit from incorporating diffusion tensor magnetic resonance imaging (DT-MRI) data from Sprague-Dawley rats to create a more accurate representation of the fiber architecture.

Finally, while isoflurane and other anesthetics can alter cardiovascular function (Loushin, 2005), isoflurane was carefully regulated at 2% (in 100% O_2_), below reported doses that drastically alter heart rate and hemodynamics (Yang et al., 2014; Pang et al., 2018). The effects on hemodynamics due to isoflurane were likely small compared with the differences due to the PAH treatment, as seen previously (Hill et al., 2014; Vélez-Rendón et al., 2019; Kwan et al., 2021).

## 5 Conclusion

The combination of finite element modeling and experimental measurements of hemodynamics reveals significant mechanical changes associated with a moderately changed functional state, as indicated by changes in ejection fraction. In this analysis we find that increased RV wall thickness, myocardial contractility and stiffness are compensatory in the initial 12 weeks post-disease induction, and these mechanisms work to alleviate the increase in wall stress and strain due to pressure overload. In the absence of these remodeling mechanisms, our model predicted that wall stress would have increased more than twenty-fold from baseline levels, which could have serious implications for myocardial perfusion and, subsequently, cardiac function.

## Data Availability

The raw data supporting the conclusion of this article will be made available by the authors, without undue reservation.
