# Supplementary material for: A computational study of right ventricular mechanics in a rat model of pulmonary arterial hypertension
Source: Front Physiol. 2024 Mar 11;15:1360389. doi: 10.3389/fphys.2024.1360389 (PMC10961401; doi:10.3389/fphys.2024.1360389)
Supplement: Supplementary file 1 [file Image1.pdf]

frontiers

Supplementary Material

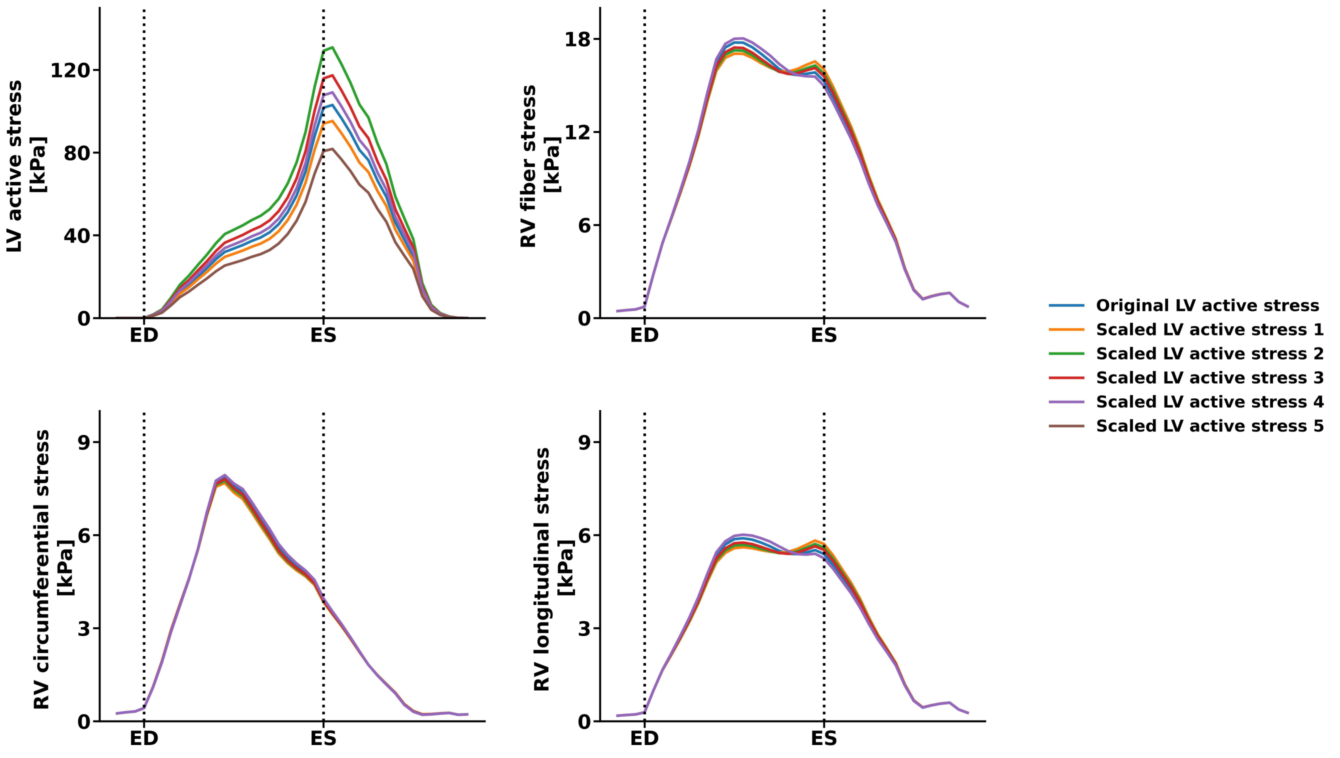

Figure S1. Sensitivity analysis of RV stress predictions to LV active stress parameter variations. This analysis is based on the control geometry, with a low resolution mesh of 5539 elements. The active stress parameter for the LV was randomly scaled between 0.7 and 1.3 times its value, resulting in five alternative time traces. The figure illustrates model predictions of fiber, circumferential, and longitudinal stress on the RV free wall based on these different time traces of the LV active stress parameter. On the x-axis we plot the normalized time over one cardiac cycle. The vertical dotted lines indicate the timings of ED and ES. Despite these variations in LV parameters, the stress predictions on the RV remain consistent. This sensitivity analysis supports our decision to employ a fixed time trace of the LV active stress parameter across all simulations. LV: left ventricle; RV: right ventricle; ED: end-diastole; ES: end-systole.
